# Comparison of articular cartilage repair with different hydrogel-human umbilical cord blood-derived mesenchymal stem cell composites in a rat model

**DOI:** 10.1186/scrt427

**Published:** 2014-03-19

**Authors:** Jun Young Chung, Minjung Song, Chul-Won Ha, Jin-A Kim, Choong-Hee Lee, Yong-Beom Park

**Affiliations:** 1 Department of Orthopedic Surgery, Samsung Medical Center, Stem Cell and Regenerative Medicine Institute, Sungkyunkwan University School of Medicine, Gangnam-gu, Irwon-dong 50, Seoul 135-710, South Korea

## Abstract

**Introduction:**

The present work was designed to explore the feasibility and efficacy of articular cartilage repair using composites of human umbilical cord blood derived mesenchymal stem cells (hUCB-MSCs) and four different hydrogels in a rat model.

**Methods:**

Full-thickness articular cartilage defects were created at the trochlear groove of femur in both knees of rats. Composites of hUCB-MSCs and four different hydrogels (group A, 4% hyaluronic acid; group B, 3% alginate:30% pluronic (1:1, v/v); group C, 4% hyaluronic acid: 3% alginate: 20% pluronic (2:1:1, v/v}; and group D, 4% hyaluronic acid:3% alginate:20% pluronic;chitosan (4:1:1:2, v/v).) were then transplanted into right knee defect in each study group (five rats/group). Left knees were transplanted with corresponding hydrogels without hUCB-MSCs as controls. At 16 weeks post-transplantation, degrees of cartilage repair were evaluated macroscopically and histologically using Masson’s Trichrome, safranin-O, Sirius red staining, and type-II collagen immunostaining.

**Results:**

Overall, group A with 4% hyaluronic acid hydrogel resulted in superior cartilage repair grossly and histologically and achieved a cellular arrangement and collagen organization pattern mimicking adjacent uninjured articular cartilage. Immunostaining and safranin-O staining also revealed that group A displayed the largest areas of type II collagen staining. Sirius red staining revealed that the organization pattern of collagen bundles was more similar to normal cartilage in group A. No evidence of rejection was found.

**Conclusions:**

The results of this study suggest that hUCB-MSCs could be used to repair articular cartilage defects *in vivo* and that hyaluronic acid is an attractive hydrogel candidate for use in combination with hUCB-MSCs.

## Introduction

Progress in cell biology and biomaterial technology has led to the therapeutic application of tissue engineering for the repair of cartilage defects. Mesenchymal stem cells (MSCs) have been well established as a potent cell source in the tissue regeneration field. In terms of chondrogenesis, MSCs originating from bone marrow (BM) and adipose tissue have been shown to require a biological environment stimulated by growth factors, which allows them to differentiate into hyaline cartilage tissues. Many previous studies have reported that MSCs originating from different tissue sources, such as BM or adipose tissue, can differentiate into chondrocytes under certain culture conditions when stimulated by various growth factors [1-4].

Human umbilical cord blood-derived mesenchymal stem cells (hUCB-MSCs) have emerged as an alternative cellular source because they offer several advantages, such as non-invasive collection, hypo-immunogenicity, superior tropism and differentiation potential [5,6]. By virtue of these properties, pre-clinical trials with hUCB-MSCs have been conducted in the contexts of Alzheimer’s disease [7], myocardial infarction [8], stroke [9] and broncho- pulmonary dysplasia (BPD) [10]. However, the effects of hUCB-MSCs on cartilage repair have not been fully evaluated.

In terms of material requirements in regenerative medicine, hydrogels have long received attention because they serve as scaffolds that provide structural integrity and bulk for cellular organization and morphogenic guidance, function as tissue barriers and bioadhesives, can deliver bioactive agents that encourage natural reparative processes, and can encapsulate and deliver cells [11]. With regard to cartilage tissue engineering, hydrogels have several advantages, such as high cell seeding efficacies, the abilities to transport nutrients and products to cells, the facility for straightforward modification with cell adhesive ligands and injectability *in vivo* as a liquid that gels at body temperature and rebuilds the three-dimensional structure [12,13].

For proper hydrogel selection in regenerative medicine, several factors need to be considered. Among them, the ability to mimick the natural cellular environment as well as clinical availability are considered the most important factors. Hydrogels need to be physically stable *in situ*, allow appropriate neovascularization and vascular remodeling, and be biodegradable to enable gradual cellular ingrowth. Furthermore, they should be biocompatible and provide an appropriate three-dimensional environment for tissue growth and cellular proliferation to promote tissue regeneration [11].

Hyaluronic acid (HA) is a high molecular weight glycosaminoglycan (GAG) that is present in all mammals and contains repeating disaccharide units composed of (β-1,4)-linked D-glucuronic acid and (β-1,3)-linked N-acetyl-D-glucurosamine. HA is particularly suitable for tissue engineering applications because of its high viscoelasticity and its space filling properties, and has already been utilized for the treatment of osteoarthritis [14]. For application in cartilage repair, HA hydrogels have been shown not only to support and maintain chondrocyte viability and phenotype when cultured *in vitro* and *in vivo* but also to support and promote the chondrogenic differentiation of MSCs [15-18]. Since the ideal hydrogel design should support the distribution of extracellular matrix by diffusion as well as maintain certain mechanical properties, preliminary studies were performed on various concentrations of HA. Pluronic, poly (ethylene oxide)-*b*-poly (propylene oxide)-*b*-poly (ethylene oxide) is a thermosensitive synthetic polymer, which can form gels above its lower critical solution temperature (LCST) due to hydrophobic interactions between its poly (propylene oxide) moieties. Chitosan is composed of relatively simple glucosamine and *N*-acetyl glucosamine units, and is a well known biocompatible material with a chemical structure similar to those of diverse GAGs in articular cartilage [13].

Several studies have described the effect of hydrogel systems for promoting cell proliferation, differentiation and gene expression for articular cartilage regeneration [19-21]. However, most of these studies were performed *in vitro*, and, to the best of our knowledge, no study has yet evaluated the feasibility and efficacy of hydrogels *in vivo* for cartilage repair using hUCB-MSCs. Since the cell delivery carrier is a key factor in the success of stem cell based cartilage regeneration and each different hydrogel has its own specific advantages and disadvantages, it is essential that the potentials of different hydrogels are investigated. Therefore, the purpose of this study was to explore the feasibility of cartilage repair using composites of hUCB-MSCs and four different hydrogels in a rat model.

## Methods

### Isolation of hUCB-MSCs

Human umbilical cord blood (hUCB) was collected from umbilical veins after neonatal delivery by an independent cord blood bank with informed consent from pregnant mothers. The hUCB-MSCs were isolated and characterized at the cord blood bank according to a previously described method [22] and donated for this animal study. Mononuclear cells were isolated by density gradient centrifugation at 550 × *g* for 30 minutes in Ficoll Hypaque (density 1.077 g/ml, Sigma, St. Louis, MO, USA). Separated mononuclear cells were then plated in α-minimum essential medium (α-MEM, Gibco BRL, Carlsbad, CA, USA) supplemented with 15% fetal bovine serum (FBS, HyClone, Logan, UT, USA), and maintained at 37°C in a humidified 5% CO_2_ atmosphere with culture medium changes twice weekly. About two weeks after plating, fibroblast-like adherent cells were observed. When monolayers of MSC colonies reached 80% confluence, cells were trypsinized (0.25% trypsin, HyClone), washed and resuspended in culture medium (α-MEM supplemented with 10% FBS).

### Animals

Twenty healthy, 18-week-old, male Sprague–Dawley rats were used in this study. Animal experiments were reviewed and approved by the Institutional Animal Care and Use Committee (IACUC) of our institution. This study also followed the National Institutes of Health Guide for Care and Use of Laboratory Animals.

### Experimental protocol

**Figure 1:**
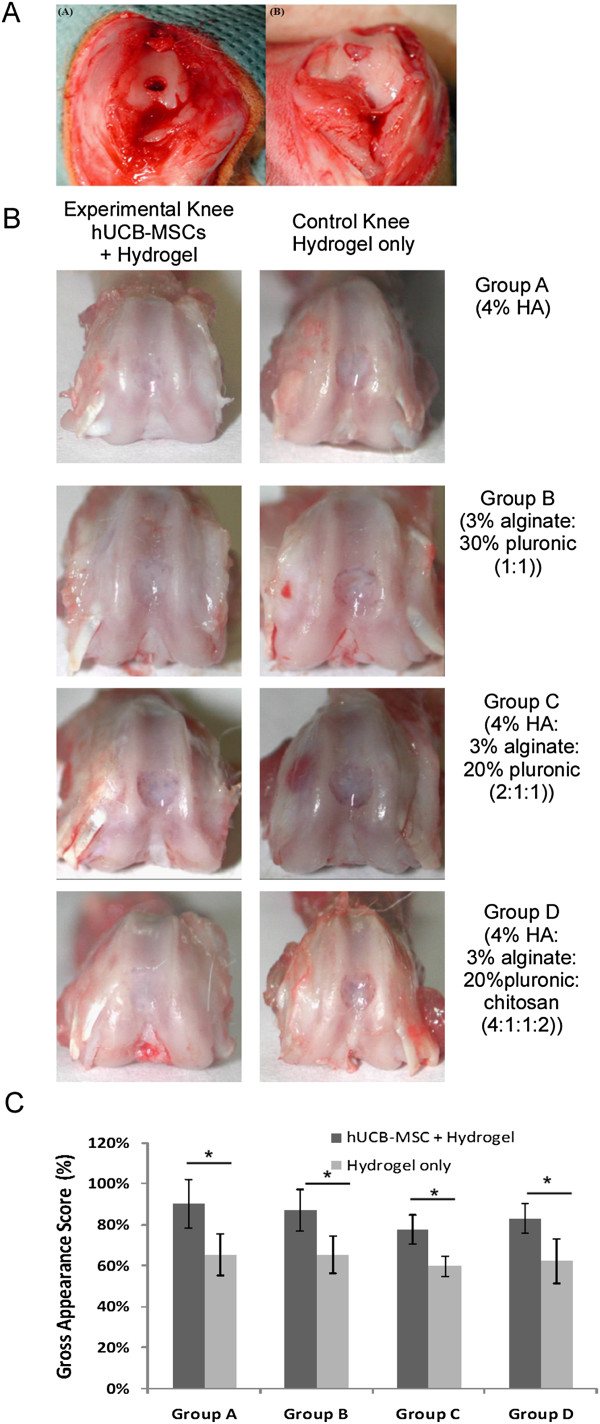
**Articular cartilage defects in a rat model and gross appearance and scoring result at 16 weeks post-transplantation. (A)** Gross photos of articular cartilage defects in a rat model. **A)** The patella was laterally dislocated and full-thickness articular cartilage defects (2 mm diameter) were created through subchondral bone in the trochlear groove in each hindlimb. B) After removing cartilage and bone debris, composites of hUCB-MSCs and different hydrogels were transplanted into defects. **(B)** Gross findings of repair tissue at articular cartilage defects in rat knees. At 16 weeks postoperatively, tissue defects in hUCB-MSC transplanted knees were repaired to almost the normal level, and border regions between repair and normal tissue were less distinct than those in corresponding control knees. No significant macroscopic differences were observed between the four different hydrogel groups. **(C)** Gross appearance scores of experimental and control knees in four different hydrogel groups (n = 5/group, *P <0.05).

The study was designed with four groups (A, B, C, D; n = 5) to evaluate four different hydrogels as well as hUCB-MSC adding values. The ‘experimental knee’ (right knee) in each group was transplanted with hUCB-MSC + hydrogel and the ‘control knee’ (left knee) in each group was transplanted with hydrogel only. The four different hydrogels were: group A, 4% hyaluronic acid; group B, 3% alginate:30% pluronic (1:1, v/v); group C, 4% hyaluronic acid:3% alginate:20% pluronic (2:1:1, v/v); and group D, 4% hyaluronic acid:3% alginate:20% pluronic: chitosan (4:1:1:2, v/v). Anesthesia was induced by inhalation of 5% ether followed by an intraperitoneal injection of xylazine 10 mg/kg and ketamine 20 mg/kg. In each case, after cleaning with 10% betadine solution, both knee joints of each rat were sterilely draped and opened using an anteromedial approach. The patellae were laterally dislocated, and full-thickness articular cartilage defects (2 mm in diameter) were created in trochlear grooves by carefully drilling in a vertical direction using a 2-mm drill. Drilling was performed 3 mm deep through subchondral bone (Figure 1A). After removing cartilage and bone debris, boundaries around the drill were trimmed using a surgical knife and washed out. The mixture of hUCB-MSCs (1.0 × 10^7^ cells/mL) and different hydrogels was then transplanted into the full-thickness defect in the experimental knee and hydrogel only into the control knee using a syringe.

**Figure 2:**
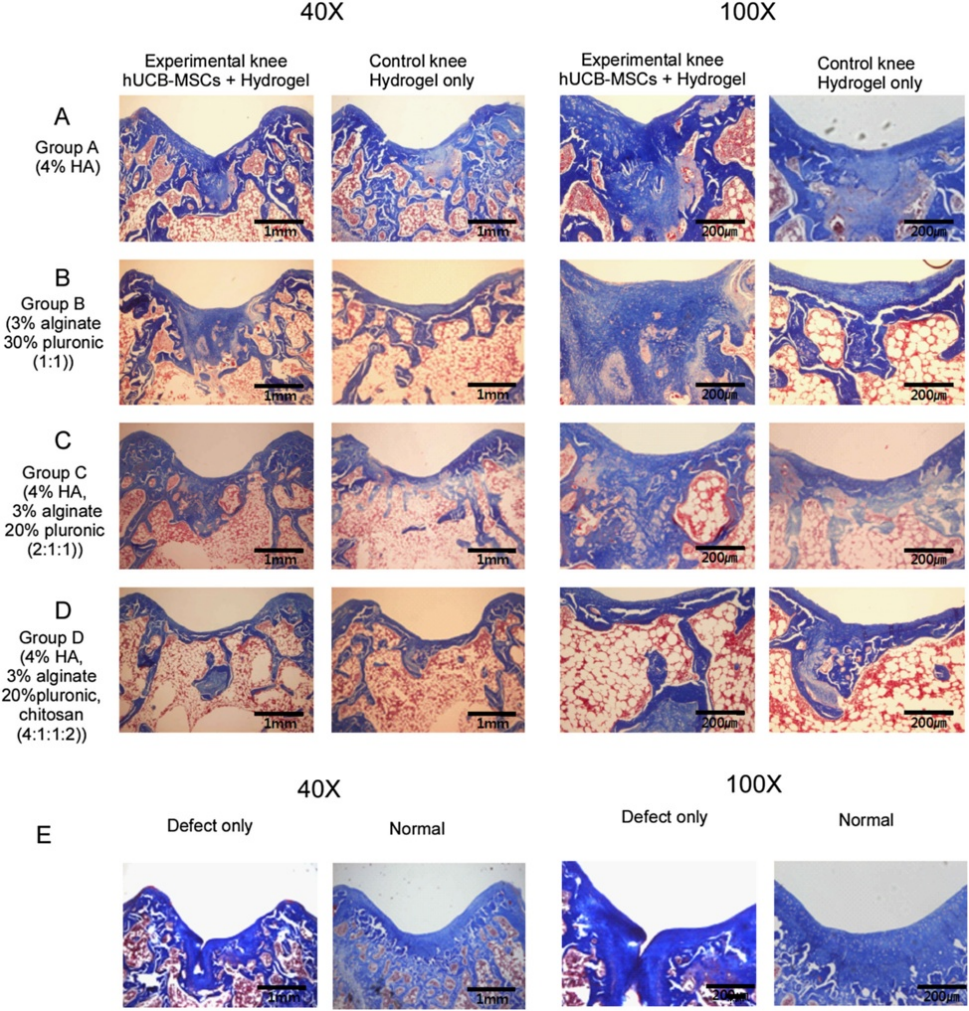
**Masson’s trichrome staining at 16 weeks post-transplantation.** Light red or pink indicates cytoplasm and dark brown to black shows cell nuclei. **(A)** Group A with hUCB-MSCs + 4% HA hydrogel composites and 4% HA only; **(B)** Group B with hUCB-MSCs + 3% alginate:30% pluronic (1:1, v/v) composites and 3% alginate:30% pluronic (1:1, v/v) only; **(C)** Group C with hUCB-MSCs + 4% hyaluronic acid:3% alginate:20% pluronic (2:1:1, v/v) composites and 4% hyaluronic acid:3% alginate:20% pluronic (2:1:1, v/v) only; **(D)** Group D with hUCB-MSCs + 4% hyaluronic acid:3% alginate:20% pluronic: chitosan (4:1:1:2, v/v) and 4% hyaluronic acid:3% alginate:20% pluronic: chitosan (4:1:1:2, v/v) only; **(E)** Defect only and normal cartilage. The transplantation of hUCB-MSCs and hydrogel composites resulted in improved articular cartilage defect repair compared with corresponding controls (hydrogels only). Of the four hydrogel compositions examined, the composite of hUCB-MSCs and HA hydrogel (group A) exhibited the most mature type of cartilaginous tissue repair with respect to cellular shape, number, lacunae formation, organization and articular contour. In group A cartilage with hUCB-MSCs and HA composites, hyaline cartilage with good column alignment of chondrocytes without cell clustering and lacunae formation were observed, which was similar to the morphology of native cartilage. In contrast, the size of chondrocyte with HA only was relatively smaller than a normal one in the mid zone. In groups B and C, moderate hypocellularity with small and flat cells in the mid zone and cell number decrease were observed. In group D with hUCB-MSCs and hydrogel, cellular alignment was reduced as well as the cell size. HA, hyaluronic acid; hUCB-MSCs, human umbilical cord blood derived mesenchymal stem cells.

As a preliminary study, the same size (2 mm diameter and 3 mm deep) defects were created and the cartilage repair with no cell and no hydrogel was observed. At 16 weeks post-transplantation, the defects showed very poor cartilage repair, such as severe lack of integration and poor positive staining (Figure 2E). The results showed that this size of articular cartilage defect in a rat joint is a critical-size defect that cannot heal by itself. Therefore, defect only without any treatment (no cell and no hydrogel, that is, defect left alone) was not included in the present study. Following implantation, patellar retinaculum and overlying soft tissues were closed in layers. Rats were allowed to move knee joints freely in their cages without restriction. An intramuscular injection of antibiotics (amikacin (Dong-A Pharmaceutical., Seoul, Korea) 12.5 mg/kg) was given immediately after implantation and once daily for three days. Clinical signs were observed daily during the study period. Animals were sacrificed at 16 weeks post-implantation to assess cartilage repair statuses. Two rats died after surgery, one in group A and the other in group B. Thus, 18 of the 20 rats that remained healthy during the study period were available for assessment. No animal was excluded for any abnormal finding.

### Gross and histological evaluations

After sacrifice, each rat was placed on an operating table, shaved around the knee joint area, and arthrotomy was performed in the same manner as during transplantation to re-inspect the intraarticular structure. After investigation of the possibilities of rejection or infection, such as severe inflammation or extensive fibrosis, or any other abnormality, the degree of cartilage repair was grossly assessed. Coloration, luster, irregularity, presence of any depression or bulging of repaired tissues in the defect area and state of the border with adjacent normal cartilage tissue were carefully examined and gross appearance was scored following a modified Carranze-Bencano scoring system [23].

For histological analysis, each specimen was stained with Masson's Trichrome (Sigma, St. Louis, MO, USA), safranin-O (Sigma), Sirius red (Sigma), and immunohistochemically stained for Type-II collagen (Millopore Corp., Billerica, MA, USA), according to the manufacturer’s instructions. Full-thickness samples (cartilage and bone) were taken from each group at 16 weeks post-implantation. Samples were fixed in 10% formaldehyde, decalcified in 10% nitric acid for three days, dehydrated in graded ethanol, and embedded in paraffin wax. Paraffin-embedded sections (4 μm) were then cut and deparaffinized.

For Masson’s trichrome staining, tissue sections were stained in Masson’s composition solution for five minutes and differentiated in 5% phosphotungstic acid for ten minutes. Tissue sections were then stained in aniline blue solution for fve minutes, and excess stain was removed by washing with 0.2% acetic acid. For safranin-O staining, sections were stained with a 0.1% safranin-O solution (Sigma).

Type II collagen was detected by immunostaining. Sections were de-paraffinized, washed with PBS and treated with 0.3% (v/v) hydrogen peroxide in methanol for 15 minutes to inactivate the endogenous peroxidases. Sections were incubated with anti type-II collagen monoclonal antibody (1:200; Millipore Corp., Billerica, MA, USA) at 4°C overnight. After washing in PBS, the sections were incubated for one hour with horseradish peroxidase (HRP) anti-mouse secondary antibody. The reactivity was detected by using the EnVision TM FLEX System-HRP (DAKO, Carpinteria, CA, USA) and developed using 3,3′-diaminobenzidine chromogen (DAB, Vector Laboratory, Burlingame, CA, USA) substrate kit according to the manufacturer’s instructions.

For Sirius red staining, slides were baked at 55°C for one hour and then hydrated using xylene and graded ethanols (100%, 95%, 85%, 75%, 60% and 50%) to distilled water. Slides were then stained for two hours in saturated picric acid containing 0.1% Sirius red F3BA (Sigma). After staining, slides were removed, washed in 0.01 N hydrochloric acid for two minutes, rapidly dehydrated using a graded alcohol series (starting at 70%) to xylene, and finally cover slipped in Permount (ThermoFisher Scientific, Waltham, MA, USA). Samples stained with Sirius red staining were examined under a polarized microscope to observe the organization patterns of collagen fibers.

Sections were analyzed semi-quantitatively using the O’Driscoll scoring system [24]. The nature of the predominant tissue (cellular morphology and safranin O staining of the matrix) was assessed by Masson’s trichrome and safranin O stainings. Structural characteristics (surface regularity, structural integrity, thickness, bonding to the adjacent cartilage), freedom from cellular changes of degeneration (hypocellularity, chondrocyte clustering) and freedom from degenerative changes in adjacent cartilage were analyzed using Masson’s trichrome stained images. All samples were scored independently by two observers and the scores were denoted by percentages. Statistical analysis was performed using the Mann–Whitney U test (SPSS, version 14) and *P* <0.05 was considered significant.

## Results

### Gross findings

At 16 weeks post-transplantation, no abnormal findings suggested rejection or infection, such as severe inflammation or extensive fibrosis in 18 rats. In groups A and B experimental knees with cells, neo-formed tissues were translucent, and a smooth and intact surface with less irregularity was observed. However, depression of repaired tissues in the defect area was often present in control knees without cells. In groups C and D, the articular surfaces of the defect site in experimental knees were relatively smoother than in control knees, and had coloration closer to that of surrounding normal cartilage than to that of control knees. Furthermore, border areas of defects were less distinct and depressions were less obvious in experimental knees than in control knees (Figure 1B). Gross appearance scoring is shown in Figure 1C and experimental knees had higher scores compared to control knees in all of the four groups (* *P* <0.05). No significant macroscopic differences were observed among the four different hydrogel groups.

### Microscopic findings

**Figure 3:**
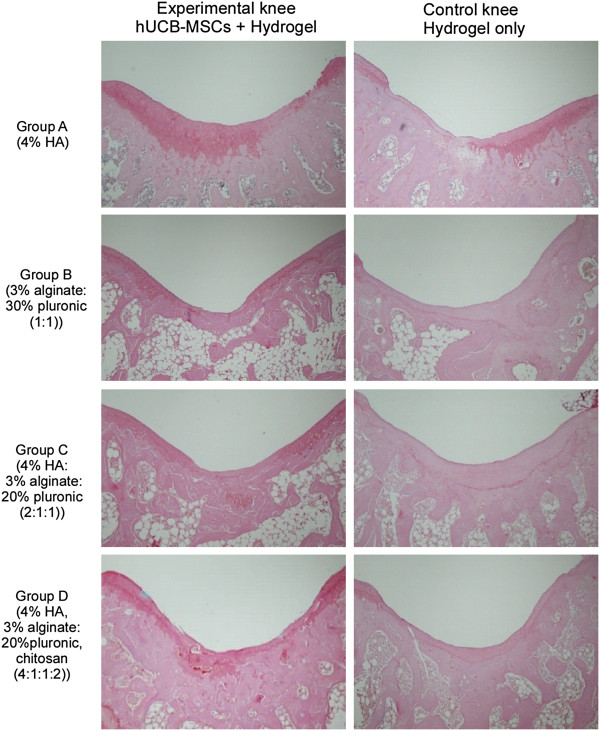
**Safranin-O staining of repair tissue at the articular cartilage defect in the rat femoral trochlear groove at 16 weeks after transplantation.** Overall, Safranin-O staining revealed more cartilaginous components in the experimental knees compared to the corresponding controls.

In Masson’s trichrome and safranin O stained specimens, experimental knees with cells in all groups were superior in the nature of the predominant tissue, structural characteristics, freedom from cellular changes of degeneration and freedom from degenerative changes in adjacent cartilage compared to control knees (Figures 2 and 3). Among the experimental knees, group A with 4% HA was superior in overall O’Driscoll score compared to control knees (Figures 2 and 3). In the group A experimental knee, a smooth and intact surface with hyaline articular cartilage with good column alignment of chondrocytes without cell clustering was observed, which is similar to the morphology of native cartilage. In the deep portion of the repair tissue, a vigorous repair process seemed to be occurring between the repair and the adjacent tissues and some tissues in the deep portion have been replaced by subchondral bone, mimicking the histological architecture of the adjacent tissues. In control knees in group A, articular contours were also restored, but a significant gap and reduced safranin O positive staining was noted in the border area stretching to subchondral bone. In addition, the size of chondrocyte was relatively smaller than normal in the mid zone. In groups B and C experimental knees, overall articular contours were restored, but the histological repair process appeared slower, more irregular and more immature than in group A. The control knees in groups B and C showed poor safranin O staining and moderate hypocellularity and cell number decrease in the defect area. In group D experimental knees, the articular contour was also restored but poor articular cartilaginous tissue formation was noted in repair tissue. Most of the defect in the deep portion had been replaced with osseous formation, indicating a rather lagging process of cartilage repair in the group D control knee. Inflammatory responses, such as cysts, were not observed in any images.

**Figure 4:**
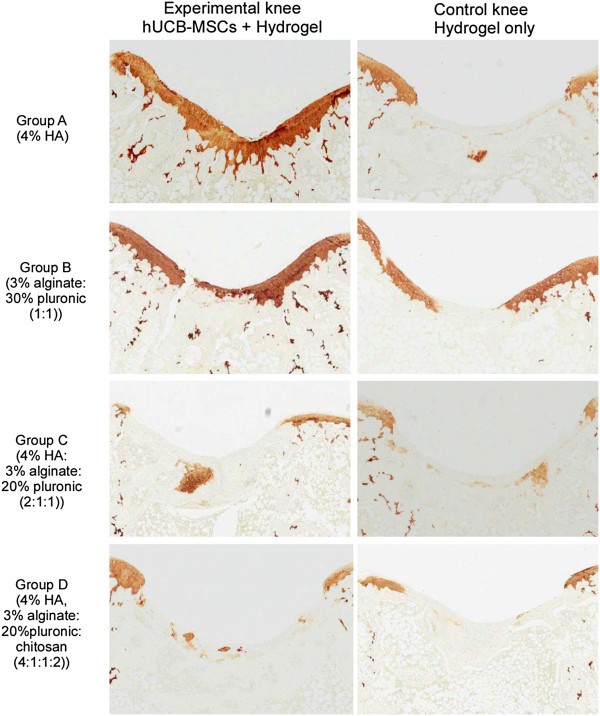
**Microscopic findings of repair tissue at the articular cartilage defect in the rat femoral trochlear groove at 16 weeks after transplantation of hUCB-MSCs and corresponding hydrogel in each group (by type II collagen immunostaining).** hUCB-MSCs, human umbilical cord blood derived mesenchymal stem cells.

With respect to immunohistochemical staining for type II collagen, specimens from experimental knees in group A showed strong diffuse staining at the defect area, indicating the production of type II collagen in the repair tissue. Specimens from groups B and C also showed moderate degrees of type II collagen immunostaining but staining was overall less evident quantitatively compared to those from group A. Specimens from experimental knees in group D showed rather pale immunostaining, indicating the presence of less articular cartilaginous tissue than in the other three groups. Overall, experimental knees in each group showed more immunostaining for type II collagen than the corresponding controls (Figure 4).

**Figure 5:**
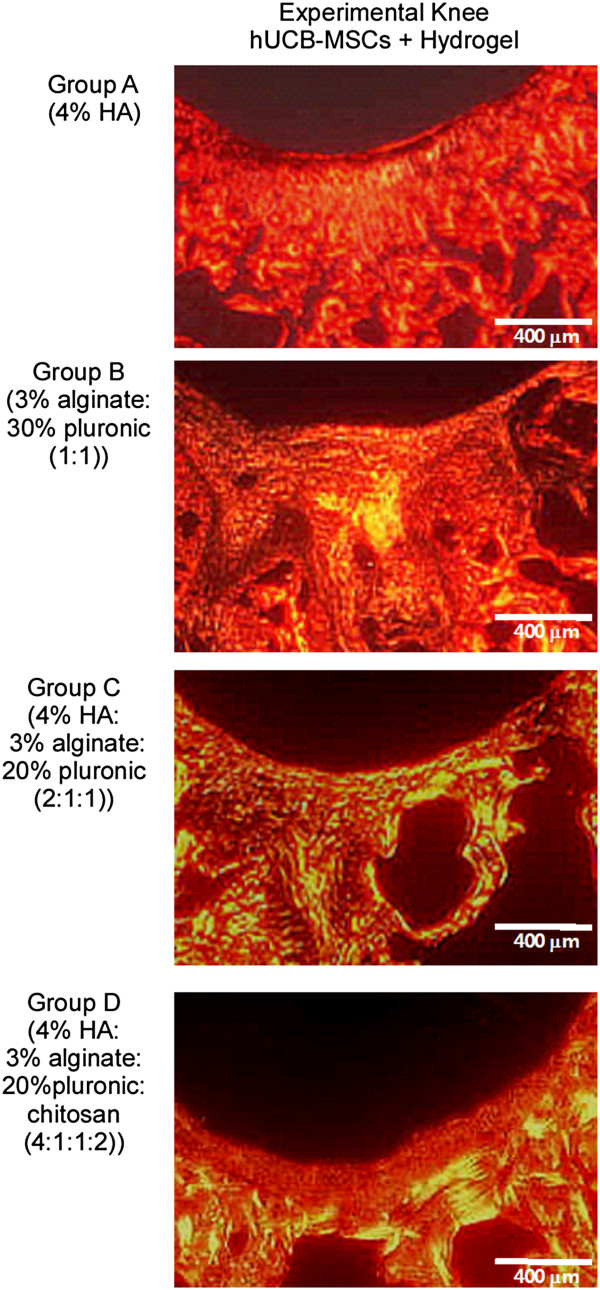
**Polarized microscopic findings of repair tissue at the articular cartilage defect in group A at 16 weeks after transplantation of hUCB-MSCs and hydrogel (by Sirius red staining).** The collagen organization pattern of repair tissue in group A was similar to that shown by surrounding normal articular cartilage with a horizontal pattern in the superficial zone and a somewhat vertical orientation in the deep zone. hUCB-MSCs, human umbilical cord blood derived mesenchymal stem cells.

Histological analysis was also performed under a polarized microscope after Sirius red staining to observe the collagen organization. The collagen fibers are indicated as bright yellow. Experimental knees from group A presented very similar collagen organization to that of the adjacent uninjured articular cartilage, which exhibited a horizontal, parallel disposition with respect to articular cartilage in the superficial zone and a vertical arrangement in the deep zone. Tissues in experimental knees from group B also showed parallel collagen disposition but this was less than that of nearby adjacent articular cartilage. In groups C and D, collagen fibers showed a parallel arrangement in the superficial zone and mid zone, which was a different pattern compared to normal cartilage (Figure 5).

**Figure 6:**
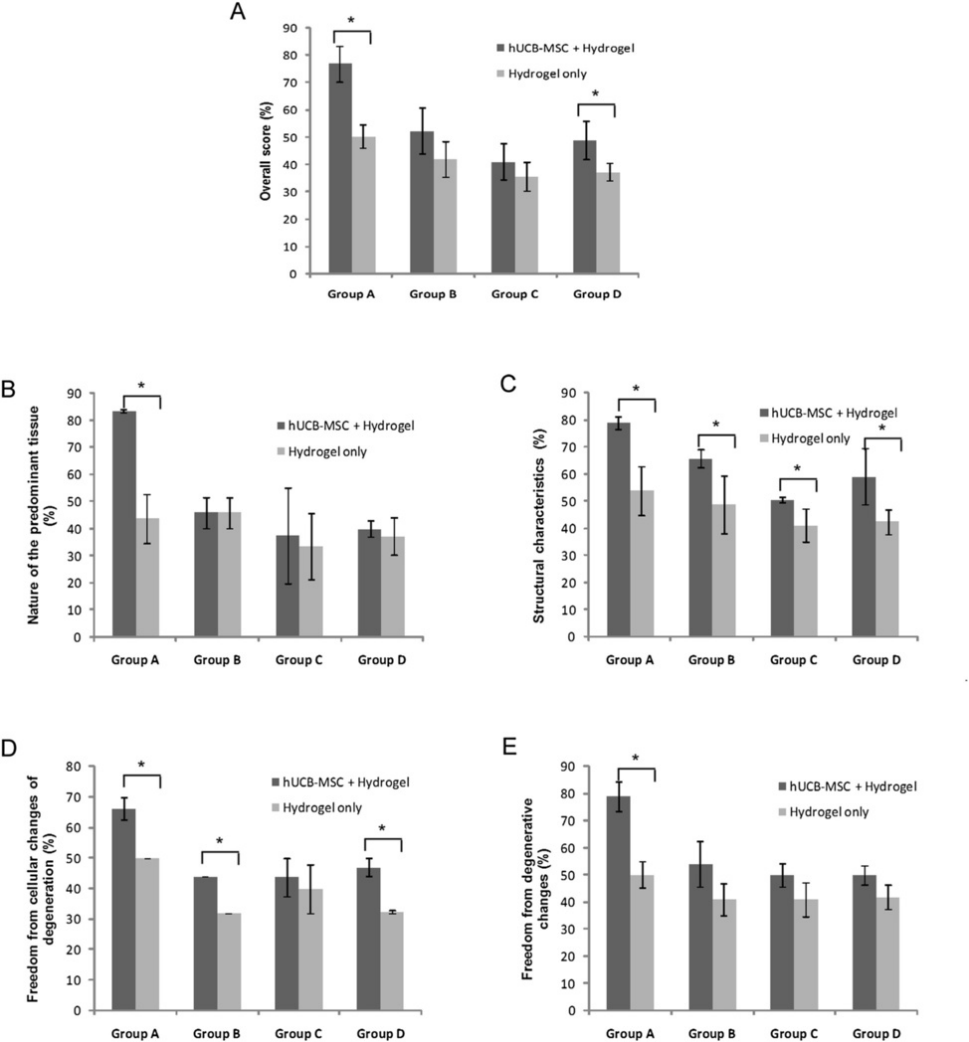
**Semiquantitative analysis of repair tissue at the articular cartilage defects between hUCB-MSC + hydrogel (experimental knee) and hydrogel only (control knee) groups. (A)** Overall score; **(B)** Nature of the predominant tissue; **(C)** Structural characteristics; **(D)** Freedom from cellular changes of degeneration; **(E)** Freedom from degenerative changes in adjacent cartilage. Statistical difference is denoted by *P <0.05.

**Figure 7:**
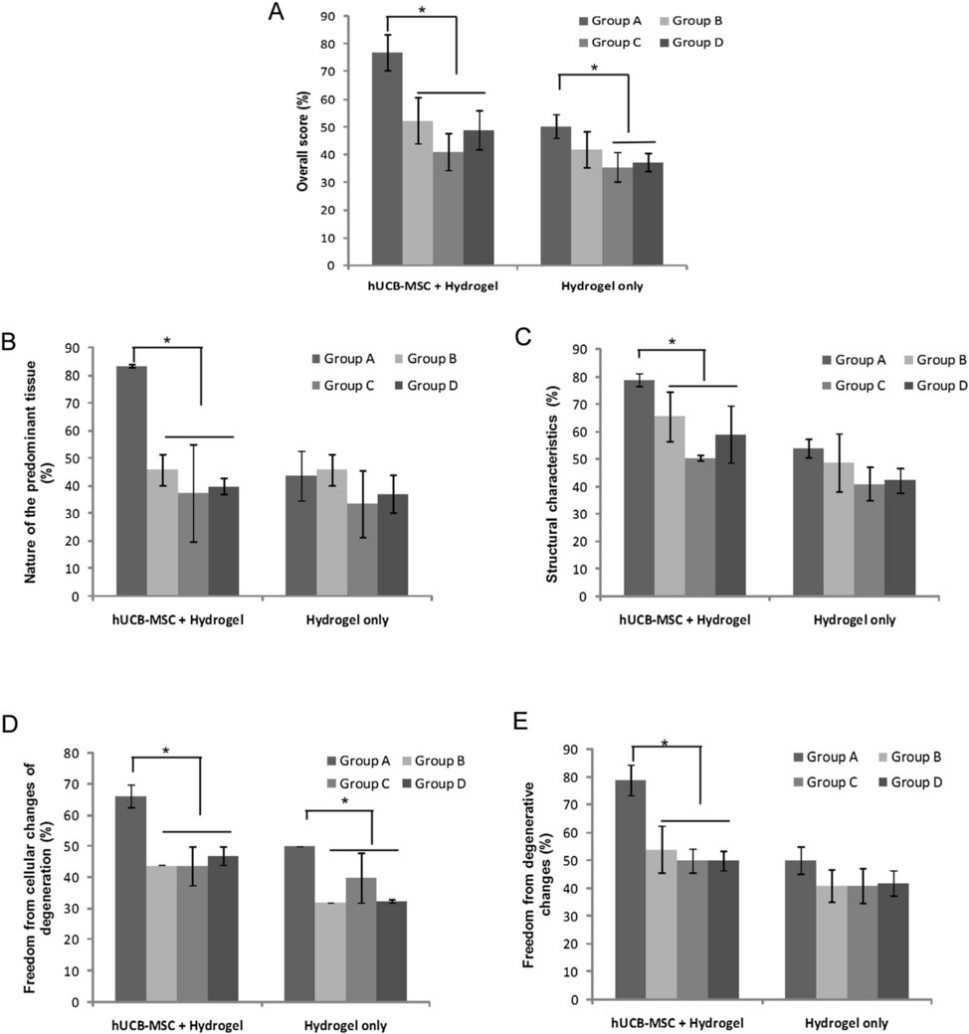
**Semiquantitative analysis of repair tissue at the articular cartilage defects among different hydrogels.** Sections were evaluated using the O’Driscoll score and denoted by percentages. **(A)** Overall score; **(B)** Nature of the predominant tissue; **(C)** Structural characteristics; **(D)** Freedom from cellular changes of degeneration; **(E)** Freedom from degenerative changes in adjacent cartilage. Statistical difference is denoted by *P <0.05.

In the semiquantitative analysis of the sections according to the O’Driscoll score, the repaired tissue in the experimental knee was histologically superior overall to that in the control knee, which showed the adding value of cells (Figure 6A). In groups A and D, the scores from the experimental knee were statistically higher in overall scores. The structural characteristics, including bonding to the adjacent cartilage with hUCB-MSC and hydrogel composites, were statistically superior compared to hydrogel only in all groups. In the parameters related to the nature of the predominant tissue and freedom from degenerative changes, the experimental knee with hUCB-MSCs had statistically higher values compared to control knee scores (Figure 6B-E). In comparisons among experimental knees with different hydrogels, 4% HA in group A was statistically higher in overall cartilage repairs including all parameters related to the nature of the predominant tissue, structural characteristics, freedom from cellular changes of degeneration and freedom from degenerative changes than the other three hydrogels (Figure 7A-E).

## Discussion

In the current study, we tried to evaluate the role of hUCB-MSCs in cartilage repair as a novel cell source. In addition, different hydrogels were evaluated to decide the most suitable one in cartilage repair. hUCB-MSC were isolated, mixed with four different hydrogels and implanted onto rat cartilage defect sites. After 16 weeks, group A with hUCB-MSCs and 4% HA showed superior cartilage repair histologically and morphologically.

Articular cartilage is highly differentiated, avascular tissue with a low self-regeneration capacity. Many researchers have attempted to increase the repair potential of damaged cartilage using cell-based therapies, such as autologous chondrocyte implantation (ACI) [25-27] or stem cell implantation [1,6,28-32]. ACI did not present the risk of immune rejection and showed significantly better histological scoring compared to the control knee [26,27]. With MACI (matrix-induced ACI), cartilage repair improvement was also reported [33]. However, their use is limited by the invasiveness of cell harvest requiring two operations, cell senescence from the long-term culture and difficulties associated with cell expansion. The biological activities of autologous cell cultures are dependent on patient age and genetic background, which may result in variable therapeutic outcomes. Therefore, allogenic applications including hUCB-MSCs offer an alternative option in cell therapy, because of their availability from cord blood banks, the non-invasive nature of collection, their suitability for immediate transplantation and their hypo-immunogenic properties [5,34]. Cartilage repair has been achieved with BM and adipose derived stem cell (ADSC) implantation [1,28-32,35,36]. However, bone formation was often observed with BM-MSCs [31,35] and decreased cartilage repairing potency was reported with ADSC-MSCs [37,38]. The present study shows no osseous transformation and no evidence of degeneration of the repaired cartilage up to 16 weeks. This could be a possible advantage of hUCB-MSCs. Although several studies have demonstrated the chondrogenic differentiation potential of hUCB-MSCs at the *in vitro* level [39-41], we could not find a report in the literature with reasonable evidence of cartilage regeneration using hUCB-MSCs *in vivo*. The results of this study suggest that hUCB-MSCs could be used for repair of articular cartilage defects *in vivo*.

In explanation of the cartilage repair mechanism with hUCB-MSCs, we could not conclude whether hUCB-MSCs regenerated the cartilaginous tissue directly or played a supportive role. Regarding the application of stem cells in regenerative medicine, several research groups have focused on the differentiation potential of stem cells, and many previous studies have demonstrated the chondrogenic potential of MSCs involved in cartilage regeneration [2,42-44]. Likewise, the repair tissue in the present study could have been produced from the chondrogenic differentiation of hUCB-MSCs. In addition to their differentiation potential, the paracrine action of MSCs is now believed to play a role in their therapeutic effects. For example, the secretion of growth factors and cytokines has been studied [3,45,46]. During the repair process, unidentified factors secreted from hUCB-MSCs may have stimulated chondrogenic differentiation or enhanced cartilage-specific extracellular matrix (ECM) synthesis by bone marrow stem cells. Yang *et al.* demonstrated possible interactions mediated by secreted proteins between human nucleus pulposus (NP) cells and MSCs [47]. Alternatively, the stimulus from MSCs in the host could modulate the therapeutic activities of transplanted MSCs. For example, secreted proteins from mature human articular chondrocytes suppressed MSC hypertrophy during chondrogenesis [4,48,49], which suggests that an interaction between hUCB-MSCs and host cells plays an essential role in cartilage regeneration. However, the key secreted factors have yet to be identified.

In addition, the anti-inflammatory and immune-modulating properties of hUCB-MSCs may also be involved in the cartilage repair mechanisms [50-52]. Singer *et al*. and Wang *et al*. demonstrated the immune modulatory function of MSCs, especially their anti-inflammatory effect, which may have affected the intraarticular microenvironment in this study and provided a suitable condition in which the transplanted hUCB-MSCs produced more relevant repair tissue even in a xeno graft trial with immunocompetent animals as in the present study [52,53]. Taken together, we believe that the mechanism of cartilage regeneration in our model is associated with chondrogenic differentiation, the paracrine action of hUCB-MSCs, and their immunomodulatory effects.

The cell concentration for the regeneration of the defect may also be an issue. In our previous study to determine the optimal cell concentration (in preparation for submission to a journal), we transplanted 0.1, 1.0 and 10 × 10^7^ cells/ml for cartilage repair *in vivo* and 1.0 × 10^7^ cells/ml showed the best result. Based upon this result, we transplanted 1.0 × 10^7^ cells/ml. Erickson *et al*. [18] encapsulated 60 × 10^6^ cells/ml as well as 20 × 10^6^ cells/ml to the methacrylated hyaluronic gels and revealed the correlation of the cell numbers with chondrogenesis and mechanical results. However, they used bovine BM-derived mesenchymal stem cells which are completely different from hUCB-MSCs. Therefore, direct comparison of these two studies in terms of the cell numbers seems to be somewhat difficult.

In the present *in vivo* study, we tried to identify a suitable and effective vehicle of cell delivery for cartilage repair. Our results indicated that cartilaginous tissue repairs were better overall in group A (the HA group) than in groups B, C or D. The superiority of the HA-based hydrogel correlates with a previous finding that HA has a beneficial effect on cartilage regeneration *in vivo*[54]. In specific, HA has biological cues to promote chondrogenesis; therefore, local progenitor cells might migrate to the HA as well. Pluronic hydrogel contains a unique thermosensitive property among the clinically available material. Therefore, we tried to evaluate pluronic hydrogel in cartilage repair. Usually a high concentration (20% to 25% w/v) of pluronic is required for *in situ* gelation, and a combination of pluronic with other *in situ* gelling- or viscosity-enhancing polymers may enable reduction in the concentration of individual polymers and also side effects without compromising the *in vitro* gelling capacity [55]. Therefore, we used 3% alginate:30% pluronic (1:1, v/v) as an *in situ* gelling polymer in group B, and 3% alginate:20% pluronic (1:1, v/v) in groups C and D combining with other polymers. Haider *et al*., showed good cell viability at this range of alginate pluronic hydrogel [56]. A few previous studies have compared different types of hydrogels to assess the influence of scaffold chemistry on chondrogenesis [15,57,58]. Chung *et al*. compared HA hydrogels with the relatively inert poly(ethylene glycol) (PEG) hydrogels and observed a 43-fold up-regulation of type II collagen of MSCs in HA over PEG in an *in vivo* culture study [15]. Wang *et al*. also examined the influence of four different hydrogels on ECM synthesis over a two-week period and the results showed that pluronic-collagen accumulated very little GAG or collagen, which seems to be in accordance with the results of the present study [58].

## Conclusions

In conclusion, the results of this study suggest that hUCB-MSCs could be used for repairing articular cartilage defects *in vivo*, and HA is an attractive hydrogel candidate for hUCB-MSCs based cartilage tissue engineering.

## Abbreviations

ACI: autologous chondrocyte implantation; BM: bone marrow; BPD: bronchopulmonary dysplasia; DAB: diaminobenzidinetetrahydrochloride; ECM: extracellular matrix; GAG: glycosaminoglycans; HA: hyaluronic acid; HRP: horseradish peroxidase; hUCB: human umbilical cord blood; hUCB-MSCs: human umbilical cord blood derived mesenchymal stem cells; LCST: lower critical solution temperature; MOD: modified O’Driscoll score; MSC: mesenchymal stem cell; NP: nucleus pulposus; PBS: phosphate-buffered saline; PEG: poly(ethylene glycol).

## Competing interests

The authors declare they have no competing interests.

## Authors’ contributions

JC collected and assembled data and drafted the manuscript. MS participated in data analysis and interpretation, manuscript writing and administrative support. CH designed and supervised the experiment, interpreted data and drafted the manuscript. JK conducted staining. CL carried out animal experiments. YP interpreted data and revised manuscript. All authors read and approved the final manuscript.
